# Novel Zinc/Silver Ions-Loaded Alginate/Chitosan Microparticles Antifungal Activity against *Botrytis cinerea*

**DOI:** 10.3390/polym15224359

**Published:** 2023-11-08

**Authors:** Marko Vinceković, Slaven Jurić, Kristina Vlahoviček-Kahlina, Katarina Martinko, Suzana Šegota, Marijan Marijan, Ana Krčelić, Lidija Svečnjak, Mislav Majdak, Ivan Nemet, Sanda Rončević, Iva Rezić

**Affiliations:** 1 Department of Chemistry, Faculty of Agriculture, University of Zagreb, Svetošimunska 25, 10000 Zagreb, Croatia; sjuric@agr.hr (S.J.); kvkahlina@agr.hr (K.V.-K.); anakrcelic15@gmail.com (A.K.); 2 Department of Plant Pathology, Faculty of Agriculture, University of Zagreb, Svetošimunska 25, 10000 Zagreb, Croatia; kmartinko@agr.hr; 3 Laboratory for Biocolloids and Surface Chemistry, Ruđer Bošković Institute, Bijenička 54, 10000 Zagreb, Croatia; ssegota@irb.hr; 4 Department of Quality Control, The Institute of Immunology, Rockefellerova 2, 10000 Zagreb, Croatia; mmarijan@imz.hr; 5 Department of Fisheries, Apiculture, Wildlife Management and Special Zoology, Faculty of Agriculture, University of Zagreb, Svetošimunska 25, 10000 Zagreb, Croatia; lsvecnjak@agr.hr; 6 Department of Applied Chemistry, University of Textile Technology, Prilaz Baruna Filipovića 28a, 10000 Zagreb, Croatia; mmajdak@ttf.hr; 7 Department of Analytical Chemistry, Faculty of Science, University of Zagreb, Horvatovac 102a, 10000 Zagreb, Croatia; inemet@chem.pmf.hr (I.N.); roncevic@chem.pmf.hr (S.R.)

**Keywords:** zinc alginate, zinc ions, silver ions, encapsulation, agriculture, *Botrytis cinerea*

## Abstract

Addressing the growing need for environmentally friendly fungicides in agriculture, this study explored the potential of biopolymer microparticles loaded with metal ions as a novel approach to combat fungal pathogens. Novel alginate microspheres and chitosan/alginate microcapsules loaded with zinc or with zinc and silver ions were prepared and characterized (microparticle size, morphology, topography, encapsulation efficiency, loading capacity, and swelling behavior). Investigation of molecular interactions in microparticles using FTIR-ATR spectroscopy exhibited complex interactions between all constituents. Fitting to the simple Korsmeyer–Peppas empirical model revealed the rate-controlling mechanism of metal ions release from microparticles is Fickian diffusion. Lower values of the release constant *k* imply a slower release rate of Zn^2+^ or Ag^+^ ions from microcapsules compared to that of microspheres. The antimicrobial potential of the new formulations against the fungus *Botrytis cinerea* was evaluated. When subjected to tests against the fungus, microspheres exhibited superior antifungal activity especially those loaded with both zinc and silver ions, reducing fungal growth up to 98.9% and altering the hyphal structures. Due to the slower release of metal ions, the microcapsule formulations seem suitable for plant protection throughout the growing season. The results showed the potential of these novel microparticles as powerful fungicides in agriculture.

## 1. Introduction

One of the biggest problems for global commercial crop and food production is plant pathogenic bacteria and fungi, which dramatically increase production costs and reduce crop establishment and productivity. Of the various natural and artificial methods of controlling plant diseases, the use of pesticides is the most widespread. The overuse of pesticides has a significant impact on the environment, food security, and human health [1,2]. Therefore, the introduction of innovative agroecological formulations is one of the most important steps in plant protection. The use of metal ions as antimicrobial agents, such as silver and zinc, seems to be the best alternative to synthetic pesticides [3].

Silver has an extremely antimicrobial effect, and its advantage is that it is more toxic to microorganisms than other metals and has lower toxicity to mammalian cells. Silver nanoparticles have become one of the most widely used alternatives as agents against various plant-damaging microorganisms [4]. In addition to being antimicrobial against various plant pathogens, silver helps plants absorb nutrients from the soil; that is, it acts as a pesticide and fertilizer [5]. The disadvantage of using silver is that it is toxic at higher concentrations and disrupts the ecosystem if released uncontrolled into the environment [6].

Zinc is an essential microelement for metabolic processes and plays a key role in the growth, development, and defense of plants. It plays a very important role in the physiological processes of plants (it participates in the construction of many enzymes and the structure of auxin, it is involved in photosynthesis and the synthesis of nucleic acids, and some proteins, etc.) and plays a key role in the response of plants to pests and diseases [7]. Various zinc fertilizers (inorganic compounds, synthetic chelates, natural organic complexes, and inorganic complexes) are used to improve plant nutrition and yields. ZnO and ZnSO_4_ are the most commonly used, but the incorporation of zinc into macronutrient formulations has become popular as it allows for a more even distribution of zinc into the soil. The availability of zinc to plants depends on the composition of the fertilizer, its interaction with the soil, and the application method. Efforts to increase the efficiency of zinc-based fertilizers are focused on the use of new technologies (nanotechnology, new polymers, and chelates) [8]. Excessive use of zinc-based fertilizers can result in various adverse effects on plants such as reduced growth, reduced photosynthesis and respiration rates, unbalanced mineral supply, and increased formation of reactive oxygen compounds.

The level of toxicity and the effects of zinc and silver ions on plants depend on the plant species, soil type, hydraulic conductivity, and availability of essential nutrients in the soil. Two review articles present the toxicity levels of zinc [9] and silver ions [10] for different plant species. The excessive use of Zn^2+^ or Ag^+^ is harmful to plants and the environment, and it is important to control the amount supplied to plants. A relatively simple solution is encapsulation in biopolymer microparticles, a technology that releases the active substance for protection or nutrition in a controlled and/or prolonged manner [11].

Biopolymers derived from biological sources are abundant in nature, biocompatible, inexpensive, and degrade without leaving toxic residues. They have many potential applications in various fields including medicine, food, and agriculture, i.e., they are widely used as matrices for the microencapsulation, viscosity modifiers, binding agents, film coating agents, disintegrating agents, solubilizing agents, emulsifying agents, suspending agents, gelling agents, etc. One of the promising applications of biopolymers is the encapsulation of bioactive agents (such as vitamins, probiotics, metals, polyphenols, microorganisms, etc.) for food and agricultural purposes. Some of the biopolymers that can be used as carriers for encapsulation of numerous bioactive compounds are alginate, chitosan, gums, gelatin, starch, and pectin [12]. The choice of biopolymer and encapsulation technique depends on the type of bioactive compound, the desired properties, and the intended application. Some of the factors that need to be considered are the physicochemical properties of the encapsulated substances, the loading efficiency and capacity, and the release kinetics and mechanism of encapsulated bioactive. The release of bioactive substances can be tailored to suit the specific application and environment [13].

In general, microparticles loaded with an active substance (microparticle formulation) protect encapsulated substances from external influences, reduce toxicity, and the amount of active substance required for application, but the most important role is the possibility of targeted and controlled release. There are two main types of microparticles: microspheres and microcapsules. A microsphere is a solid matrix particle, while a microcapsule has an inner core and outer shell. Chitosan and alginate are two important biopolymers with great potential in enhancing stability, durability, and effectiveness of encapsulated bioactive agents under field conditions, benefiting crop growth and yield [12]. Chitosan, a biopolymer derived from chitin, has an important application in agriculture due to its multiple effects. Alone or in combination with other biopolymers, it can be used as a matrix for the encapsulation of bioactive agents and can form films and nanoparticles. The important property of chitosan is its antibacterial and antifungal activity. While it does not directly kill pathogens, it enhances plant resilience by triggering defense mechanisms and interfering with microbial growth [14,15]. However, the insolubility of chitosan in neutral or alkaline conditions suppresses its action on phytopathogens [14].

Alginate, a natural biopolymer derived from brown algae, offers several advantages for encapsulation compared to other biopolymers. Alginate offers practical and profitable solutions for enhancing the effectiveness of bioactive agents in agriculture by providing protection and stability, ensuring constant availability of beneficial compounds to plants over time. The advantages of alginate as a carrier are nontoxicity, simplicity, biocompatibility, biodegradability, low cost, and easy formation of gel microparticles at mild processing conditions [16]. A common method to improve the sustainable release of an active agent from alginate microspheres is to coat them with oppositely charged biopolymer (such as chitosan) to form microcapsules. The polyelectrolyte layer on the surface of a microparticle provides an additional barrier that slows the transport of the bioactive to the surrounding solution [17,18].

This work aimed to produce and study the properties of (i) zinc alginate microparticles and (ii) zinc alginate microparticles loaded with silver ions as potential formulations for plant protection. The role of zinc ions is twofold: (i) it is an alginate gelling cation forming microspheres by crosslinking alginate chains and (ii) it plays a very important role in plant response to pests and disease. The antifungal potential of the new formulations was evaluated against the fungus *Botrytis cinerea* as a model pathogen [19].

## 2. Materials and Methods

### 2.1. Materials

Low-viscosity sodium alginate (ALG) (CAS Number: 9005-38-3; Brookfield viscosity 4–12 cps (1% in H_2_O at 25 °C)) was purchased from Sigma-Aldrich (St. Louis, MO, USA). Chitosan (CS) of low molecular weight (CAS RN: 9012-76-4, molecular weight: 100,000 to 300,000) was obtained from Acros Organic (Morris Plains, NJ, USA). ZnSO_4_ × 7H_2_O and AgNO_3_ were purchased from Gram-mol d.o.o. (Zagreb, Croatia). All other compounds were of analytical quality and used as they arrived, with no additional purification.

#### 2.1.1. Microparticle Preparation

Two kinds of microparticles were prepared: microspheres and microcapsules. Microspheres were prepared by ionic gelation (alginate gelation with zinc ions), whereas microcapsules were prepared in two steps by ionic gelation (alginate gelation with zinc ions) and polyelectrolyte complexation (chitosan with alginate) on the microsphere surface.

Ionic gelation involves the preparation of zinc alginate microspheres (abbreviated ALG/Zn) by dripping 500 mL of sodium alginate solution (1.5%) into equal volume of ZnSO_4_ solution (1 mol dm^−3^) or a mixture of ZnSO_4_ (1 mol dm^−3^) and AgNO_3_ (0.0032%) to prepare zinc/silver alginate microspheres (abbreviated ALG/(Zn+Ag)) through the encapsulator nozzle size of 450 μm at 300 Hz vibration frequency and 60 mbar pressure (Encapsulator Büchi-B390—BÜCHI Labortechnik AG, Flawil, Switzerland). Microspheres (ALG/Zn or ALG/(Zn+Ag)) were formed in the crosslinking solution under mechanical stirring. Microspheres were stirred for 30 min to allow full penetration of the ions to the core of the microspheres. Microspheres were then washed several times with deionized and sterilized water, filtered through a Büchner funnel, and stored at 4 °C until further studies.

Microcapsules were prepared by coating microspheres with a polyelectrolyte layer formed by the complexation of alginate and chitosan. Chitosan was solubilized in acetic acid solution (0.5% CS (*w*/*v*) in 1.0% CH_3_COOH (*v*/*v*)). Washed microspheres (ALG/Zn or ALG/(Zn+Ag)) were dispersed in 500 mL of chitosan solution and subjected to mechanical stirring. The contact time between microspheres and chitosan solution was about 30 min in order to give chitosan time to form a layer around microsphere. The obtained microcapsules (abbreviated CS/(ALG/Zn) or CS/(ALG/(Zn+Ag))) were filtered, washed with deionized and sterilized water, and stored at 4 °C until further studies.

#### 2.1.2. Test Organism

An autochthonous isolate of *B. cinerea* isolated from strawberry fruit with symptoms of gray mold was used. The isolate was identified morphologically and molecularly. A pure culture of the isolate was inoculated onto the medium, incubated in chamber climate conditions until use (22 °C, dark), (Potato dextrose agar, Liofilchem, Roseto degli Abruzzi, Italy) and incubated in chamber climate conditions until use (22 °C, dark).

### 2.2. Methods

#### 2.2.1. Chemical Fingerprinting by Fourier Transform Infrared Spectroscopy Coupled with Attenuated Total Reflectance (ATR)

Samples were analyzed by Fourier transform infrared spectroscopy (FTIR) coupled with Attenuated Total Reflectance (ATR) recording technique. FTIR-ATR spectra of the samples were acquired using the Cary 660 FTIR spectrometer (Agilent Technologies, Palo Alto, CA, USA) and the Golden Gate single-reflection diamond ATR accessory (Specac, Fort Washington, MD, USA). Spectra were recorded in the mid-infrared region (spectral range: 4000–400 cm^−1^) and in transmission mode. Before the spectral analysis, samples were pulverized into finer homogenates with a porcelain mortar. Approximately 10 mg of the sample was pressured on a diamond ATR plate using a self-leveling sapphire anvil to obtain the FTIR-ATR spectrum of a thin uniform layer of each sample. Two replicate spectra (32 scans/spectrum) of each sample were recorded using different aliquots. Spectra were acquired at a nominal resolution of 4 cm^−1^ and at room temperature (24 ± 2 °C). Raw spectral data were stored and pre-analyzed using the Agilent Resolutions Pro version 5.3.0 software package (Agilent Technologies, Palo Alto, CA, USA), while further spectral data analysis and processing were carried out using optical spectroscopy software Spectragryph (version 1.2.16.1) and Origin 8.1 (Origin Lab Corporation, Northampton, MA, USA).

#### 2.2.2. Microscopic Observations

(a) Microscopic analysis of microparticle size, surface morphology, and topography

Microparticle size, shape, surface morphology, and topography were analyzed by several microscopic techniques: (i) light microscopy (LM) (Leica MZ16a stereo microscope, Leica Microsystems Ltd., Balgach, Switzerland), (ii) scanning electron microscope (SEM) (FE-SEM, model JSM-7000F, Jeol Ltd., Tokyo, Japan), and (iii) atomic force microscope (AFM) (Bruker Billerica, Billerica, MA, USA). All sample preparations for microscopic observation were performed at room temperature.

The average diameters of wet and dry microparticles were determined by light microscopy using Olympus Soft Imaging Solutions GmbH, (version E_LCmicro_09Okt2009). Twenty microparticles were randomly selected from batches, produced in triplicate, in order to determine the size distribution.

Microparticles for SEM analysis were placed on high-conductive graphite tape. FE-SEM was connected to an EDS/INCA 350 (energy dispersive X-ray analyzer) manufactured by Oxford Instruments Ltd. (Oxford, UK). ImageJ software (vij153) was used to determine the size of pores on a microparticle surface.

The samples for AFM imaging were prepared by the deposition of a suspension of microparticles on the mica substrate. Microparticles were rinsed three times with 50 mL MiliQ water to remove the remaining impurities. All AFM imaging was performed on three different regions of each microparticle to ensure consistency in the results obtained.

(b) Microscopic analysis of the potential antifungal effect on the microstructures of *B. cinerea*

To investigate the effect of microparticles on the microstructures of *B. cinerea*, a light microscope and a stereomicroscope were used to examine the structural change of fungal samples after treatment with microparticles. Microscopic preparations containing pathogen hyphae from the control Petri dish, pathogen hyphae from test variants with microparticles, and the observed structural changes were photographed. Based on the observed structural changes in the pathogen’s hyphae, the antifungal effect of the microparticles was quantified. Photographs were processed with ImageJ, open-source software (US National Institutes of Health, Bethesda, MD, USA) according to the modified method of Guzmán et al. [20]. Based on the mean values (cm^2^) of the mycelial surface of *B. cinerea*, the inhibition index (I) was calculated.

#### 2.2.3. Encapsulation Efficiency and Loading Capacity

(a) Encapsulation efficiency (EE)

The encapsulation efficiency was calculated from the amount of total Zn or Ag ions added initially (c_tot_) and the content of Zn and Ag ions of dry beads (c_load_) by the method of Xue et al. [21]. The encapsulation efficiency was expressed as the percentage of total available Zn or Ag ions (c_tot_) and calculated by the equation:

EE = (c_load_/c_tot_) × 100 (1)

where c_load_ = c_tot_ − c_f_, and c_f_ is a concentration of Zn or Ag ions in the filtrate determined by the Agilent 7900 ICP-MS (Agilent Technologies, Singapore).

(b) Loading capacity (LC)

Wet microparticles were air-dried at room temperature for several days until all the liquid evaporated. The Zn or Ag content was determined by dissolving 1 g of microparticles in 10 mL of a mixture of 0.2 mol dm^−3^ NaHCO_3_ and 0.06 mol dm^−3^ Na_3_C_6_H_5_O_7_ × 2H_2_O at pH 8 [22]. The resulting solution was filtered and the concentration of Zn or Ag ions in the filtrate was determined using the Agilent 7900 ICP-MS (Agilent Technologies, Singapore). The loading capacity expressed as the amount of Zn or Ag ions per 1 g of microparticles was calculated by the equation:

LC = ((c_Zn or_ c_Ag_) × V/1000)/w_c_), (2)

where c_Zn or_ c_Ag_ is a concentration of Zn or Ag ions in the filtrate, V is the volume of the sample, and w_c_ is the weight of the capsules used.

#### 2.2.4. Swelling Degree (S_w_)

To avoid the influence of electrolytes from the buffer, the swelling degree (S_w_) was determined on microparticles dispersed in deionized water. The samples were prepared by dispersion of dry microparticles (0.1 g) in a glass vial containing 10 mL of deionized water and allowed to swell at room temperature for 3 h. The wet weight of the swollen microcapsules was determined by blotting them with filter paper to remove moisture adhering to the surface, immediately followed by weighing [23]. The degree of swelling (S_w_) was calculated using the equation:

S_w_(%) = ((w_t_ − w_0_)/w_0_) × 100, (3)

where w_t_ is the weight of the swollen microparticles and w_0_ is their initial weight.

#### 2.2.5. In Vitro Active Agents Release

In vitro release of Zn or Ag ions from microparticles was studied by dispersing 1 g of microparticles in 10 mL of deionized water and left to stand without stirring during experiments at room temperature. At appropriate time intervals, the dispersion was stirred for 60 s, aliquots were withdrawn, and the concentration of Zn or Ag ions was determined by Agilent 7900 ICP-MS (Agilent Technologies, Singapore). The fraction of released Zn (f_Zn_) or Ag (f_Ag_) ions was calculated using the following equation:

f = (R_t_/R_tot_), (4)

where R_t_ is the concentration of Zn or Ag ions released in solution at the appropriate time and the concentration of Zn or Ag ions loaded in the microparticles.

#### 2.2.6. Testing the Antifungal Effect of Microparticles on the Growth of *B. Cinerea*

The samples for testing were prepared by inoculation with *B. Cinerea* into the previously poured PDA medium using a micellar disk (Ø 5 mm) and incubated in a climate chamber for 5 days, at 22 °C, in the dark. Antifungal tests were performed by modified agar dilution method [24]. According to the method protocol, each type of microparticle in a volume of 1 mL and 2 mL was applied to the liquid PDA substrate (10 mL). The solution of microparticles and the nutrient medium were mixed to evenly distribute the microparticles within the substrate, after which the substrate was allowed to solidify. With a circular cutter, micellar disks (Ø 5 mm) of *B. Cinerea* were cut and grafted onto the medium, in the center of the Petri dish. The control Petri dishes contained only the pathogen disc, without microparticles. The inoculated Petri dishes were wrapped in parafilm and incubated in a climate chamber at 22 °C in the dark. All tests were carried out in triplicate.

The effectiveness of the microparticle treatment was evaluated after 5 days by photographing the control and test Petri dishes. Based on the mean values of the mycelial surface, the inhibition index (I) was calculated, which quantified the antifungal effect of the microparticles.

#### 2.2.7. Statistical Analysis

All measurements were carried out in triplicate. Results are presented with mean values and standard deviations. Data corresponding to a normal distribution were analyzed by one-way analysis of variance (One Way ANOVA), and differences between treatments were evaluated by the Tukey test (*p* ≤ 0.05) in the statistical program SPSS, version 27 (IBM SPSS Statistics IBM, Corp., New York, NY, USA, 2023).

## 3. Results and Discussion

The results are presented and discussed in two sections. In the first section, the physicochemical properties of zinc alginate microspheres and microcapsules prepared without and with silver ions are evaluated and compared. In the second part, the antifungal effects of prepared microparticles against the model pathogen, *B. cinerea*, are described.

### 3.1. Evaluation of Microparticle Physicochemical Properties

#### 3.1.1. Identification of Interactions between Microparticle Constituents

**Figure 1:**
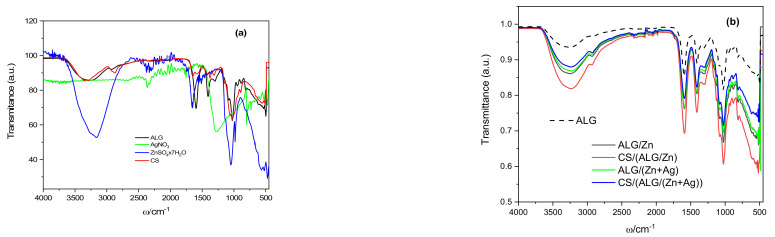
FTIR spectra of (**a**) sodium alginate (ALG), silver nitrate (AgNO_3_), zink sulfate heptahydrate (ZnSO_4_ × 7H_2_O) and chitosan (CS) and (**b**) ALG/Zn (black line) and CS/(ALG/Zn) (red line), ALG/(Zn+Ag) (green line), CS/(ALG/(Zn+Ag)) (blue line). The sodium alginate (ALG) is shown for comparison (dashed black line).

The infrared spectra of the initial components of sodium alginate, chitosan, zinc sulfate, and silver nitrate were recorded (Figure 1a) and compared with the microparticle spectra (Figure 1b). Despite the complexity of the spectra because of the overlapping of different absorption bands, as well as the coupling of different vibrations, valuable information was obtained about the molecular interactions of microparticle constituents. In the spectra analyses, more attention is focused on those absorption bands corresponding to the characteristic molecular vibrations where changes occur due to the alginate gelation. The most noticeable changes are observed in the area of the functional groups of hydroxyl and carboxyl.

The spectrum of the sodium alginate shows a strong broad band assigned to the stretching modes of the hydroxyl groups (–OH) with a peak at 3300 cm^−1^ and a shoulder at 3198 cm^−1^. Stretching vibrations of weak intensity observed at 2900 cm^−1^ correspond to –CH_2_ groups and very sharp stretching at 1595 cm^−1^ and medium sharp stretching at 1405 cm^−1^ correspond to vibrations of asymmetric and symmetric carboxyl groups (COO^−^). A weak broad CO stretching can be observed at 1295 cm^−1^, while bands at 1026 cm^−1^ are characteristic of polysaccharides [25].

The spectrum of chitosan is characterized by a broad band occurring between 3291–3610 cm^−1^ due to the stretching vibrations of the –OH groups that overlapped with the stretching vibration of the −NH_2_ groups. Vibrations at 2925 cm^−1^ (peak of lower intensity) and 2875 cm^−1^ (peak of higher intensity) are assigned to asymmetric and symmetric modes of CH_2_ vibrations, respectively. Characteristic bands of N-acetyl groups: C=O stretching (amide I), −NH bending (amide II), and C-N stretching (amide III), are arising at wave numbers 1645, 1550 and 1325 cm^−1^ [26]. The medium intensity peak at 1373 cm^−1^ belongs to the symmetric CH_3_ deformation, while the vibrations in the region 1190–920 cm^−1^ belong to the C-N stretching and overlapping vibrations from the carbohydrate ring [27].

The spectrum of silver nitrate has several peaks at 3328, 2129, and 1635 as well as a broad high intensity at 1300 cm^−1^ due to the stretching vibration of the N=O bond in NO_3_^−^ [28]. Due to the presence of water, the spectrum of zinc sulfate heptahydrate shows a broad band around 3100 cm^−1^ and a narrower band at 1657 cm^−1^ occurring due to O–H stretching vibrations and H–O–H deformation vibrations, respectively. The peaks observed at 1100, 983, and 612 cm^−1^ correspond to the stretching vibration of sulfate groups [29].

The FTIR-ATR spectra of the microparticles are presented in Figure 1b. The presence of cations in the alginate matrix causes the most significant changes in the area of alginate functional groups, hydroxyl (OH) and carboxyl (COO^−^), indicating that the interactions of microparticle constituents are mainly hydrogen bonds and electrostatic interactions [11]. Compared to the sodium alginate spectrum, the spectrum of zinc alginate shows a more intense and wider stretching band of hydroxyl groups (–OH), which indicates the formation of new hydrogen bonds. More intense, somewhat broader bands of asymmetric (at 1589 cm^−1^) and symmetric (at 1409 cm^−1^) (COO^−^) carboxyl groups are the result of interactions with Zn^2+^ ions and crosslinking by binding to carboxyl groups of guluronic and mannuronic acids, in contrast to Ca^2+^ ions that bind mainly to guluronic carboxyl groups [30]. The structure of the three-dimensional network of zinc alginate is looser and contains larger amounts of water than calcium alginate.

The mechanism of ion-induced crosslinking of alginate chains is based mainly on the interaction of multivalent cations and carboxyl groups. According to the quantum chemical computational method, alkaline earth cations form ionic bonds with alginate chains, while transition metal ions form complexes with covalent coordination bonds [31]. The binding of zinc ions has been attributed, by some authors, to an exclusive covalent coordination bond with carboxyl groups [32,33]. However, although Zn^2+^ is a transition metal (but with an ionic radius comparable to alkaline earth metals), the analysis of the main features of zinc hydrogels showed more similarities to alkaline earth hydrogels. It was concluded that the bond of zinc with alginate, although quite weak, is predominantly ionic [33].

The spectrum of ALG/(Zn+Ag) shows slightly lower intensities of the characteristic peaks without significant shifts. A somewhat narrower and less intense absorption band of hydroxyl groups around 3300 cm^−1^ and reduced intensities of the peaks attributed to carboxyl groups indicate slightly weakened hydrogen bonds and electrostatic interactions compared to ALG/Zn. The lower intensity of the broad band assigned to the hydroxyl groups implies the breaking of some of the inter- and intramolecular hydrogen bonds in ALG/(Zn+Ag), possibly due to the formation of a complex between Ag^+^ and oxygen atoms from O-H groups [34]. Furthermore, from the slight differences in the spectra of zinc alginate microparticles and those with encapsulated silver, it was not possible to unambiguously detect the formation of a bond between sodium alginate and silver ions. Lin et al. [35] attributed the interaction of Ag^+^ ions with the sodium alginate matrix to van der Waals interactions, while Zhang et al. [36] showed that Ag^+^ can also react electrostatically with anionic alginate, that is, under certain conditions, it is possible to gel alginate by ionic crosslinking with monovalent Ag^+^ cations, similar to crosslinking with Ca^2+^ cations.

A significant increase in the intensity and broadening of the absorption band around 3300 cm^−1^ and an increase in the intensities of vibrations at 1406, 1292, and 1020 cm^−1^ in the spectrum of zinc alginate coated with a layer of chitosan (CS/(ALG/Zn)) are the result of the formation of hydrogen bonds due to the complexation of Zn^2+^ ions with the amino and hydroxyl groups of chitosan [37,38,39]. The lack of other characteristic chitosan bands in the spectra can be explained by the complexation of sodium alginate and chitosan [11].

All characteristic band intensities in the spectrum of CS/(ALG/(Zn+Ag)) are slightly reduced compared to those of ALG/(Zn+Ag). The broad band around 3300 cm^−1^ is mainly attributed to N-H stretching vibrations (amide A), although in that region the N-H and O-H stretching vibrations overlap [40]. Transmission reduction in this region shows that Ag^+^ is bound to N-H groups. Other changes observed in the wavenumbers (shifts from 1585 to 1571 cm^−1^, 1406 to 1392 cm^−1^, and from 1020 to 1045 cm^−1^) are related to the bending of N-H groups, by stretching of C-N groups and N-H oscillating deformation. All these changes indicate that Ag^+^ ions bind to nitrogen atoms and thus reduce the intensity of vibrations of N-H bonds [41].

The comprehensive analysis of the infrared spectra provided insightful details into the molecular interactions and structural changes of the sodium alginate, chitosan, zinc sulfate, and silver nitrate when formulated as microparticles. The significant alterations in the absorption bands, especially within the regions associated with hydroxyl and carboxyl groups, reinforced the understanding of the interactions, including hydrogen bonding and electrostatic interactions, prevalent among the microparticle constituents. This deepened understanding of the ion-induced crosslinking and associated bonding dynamics paves the way for fine-tuning the microparticle properties for desired applications.

#### 3.1.2. Size, Surface Morphology, and Topography of Microparticles

Generally, the size and uniformity of microparticles are determined mainly by the viscosity of the alginate solution, the diameter of the nozzle, the solution flow rate, and the distance between the point from a nozzle to the gelling bath [42]. Under our experimental conditions, the microparticles prepared were mostly spherical and light brown. The surface of the wet microspheres was smooth, while the surface of the wet microcapsules was slightly rippled. The addition of silver and chitosan increased the size (Table 1). By lyophilization, the sphericity of the microparticles was lost, and the surface characteristics were modified becoming irregular and wrinkled. Their sizes are reduced and approximately two times smaller than those of wet microcapsules (Table 1).

**Figure 2:**
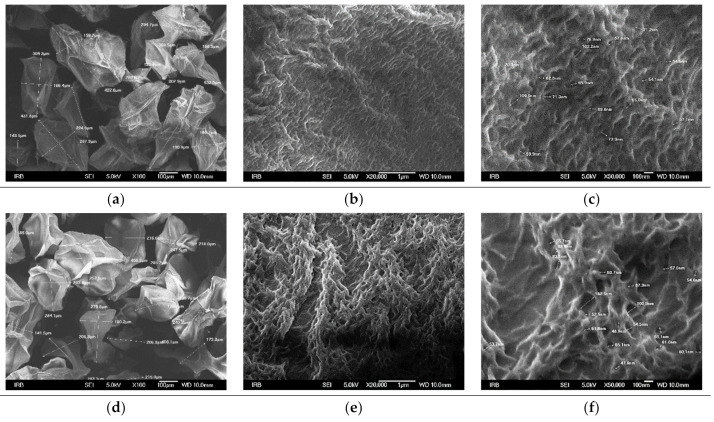

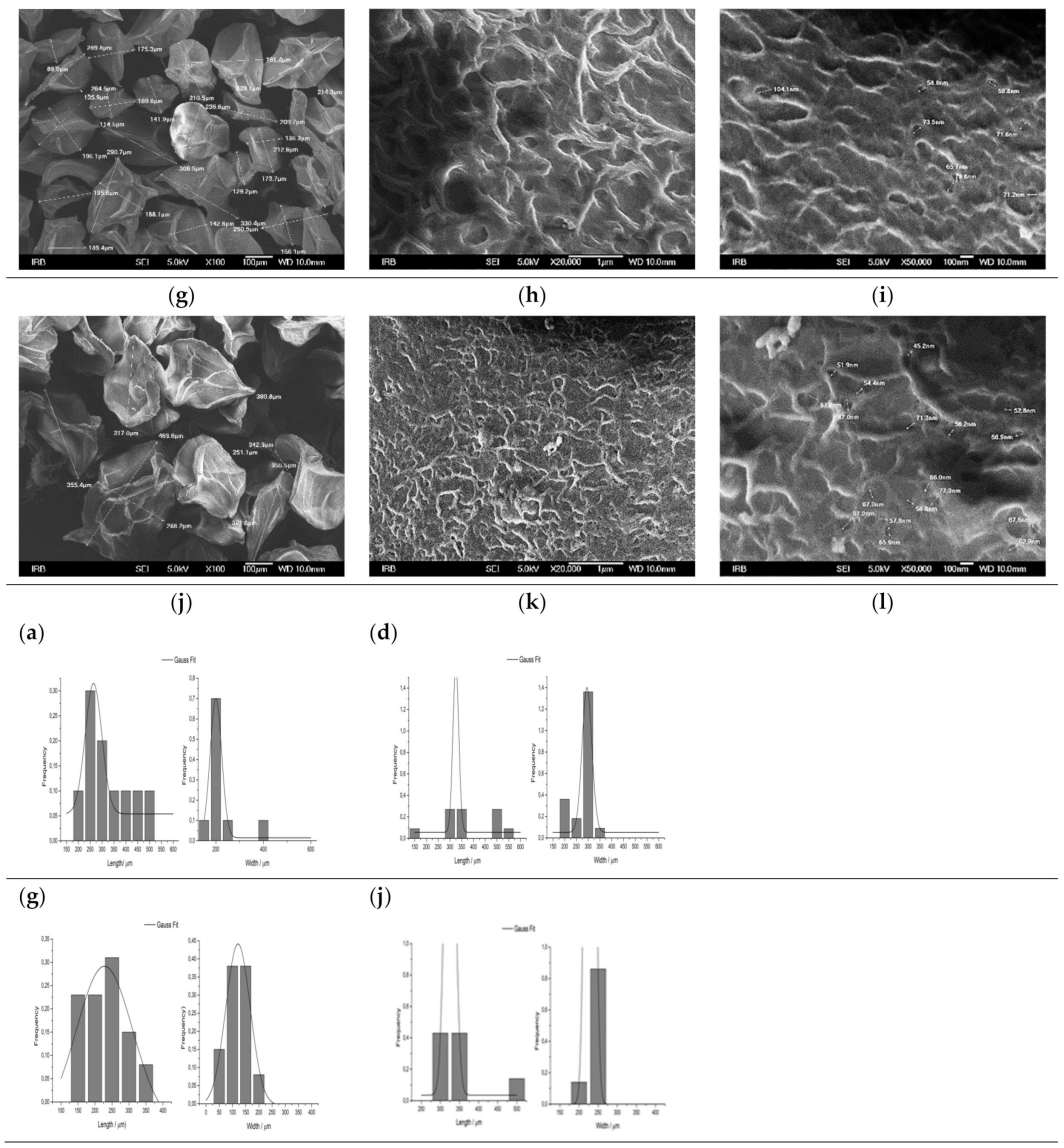
SEM images of ALG/Zn (**a**–**c**), CS/(ALG/Zn) (**d**–**f**), ALG/(Zn+Ag) (**g**–**i**) and CS/(ALG/(Zn+Ag)) (**j**–**l**) microparticles under different magnifications. Bars are indicated. The size distribution histograms for samples are obtained from SEM images (**a**,**d**,**g**,**j**) and presented on the bottom.

**Figure 3:**
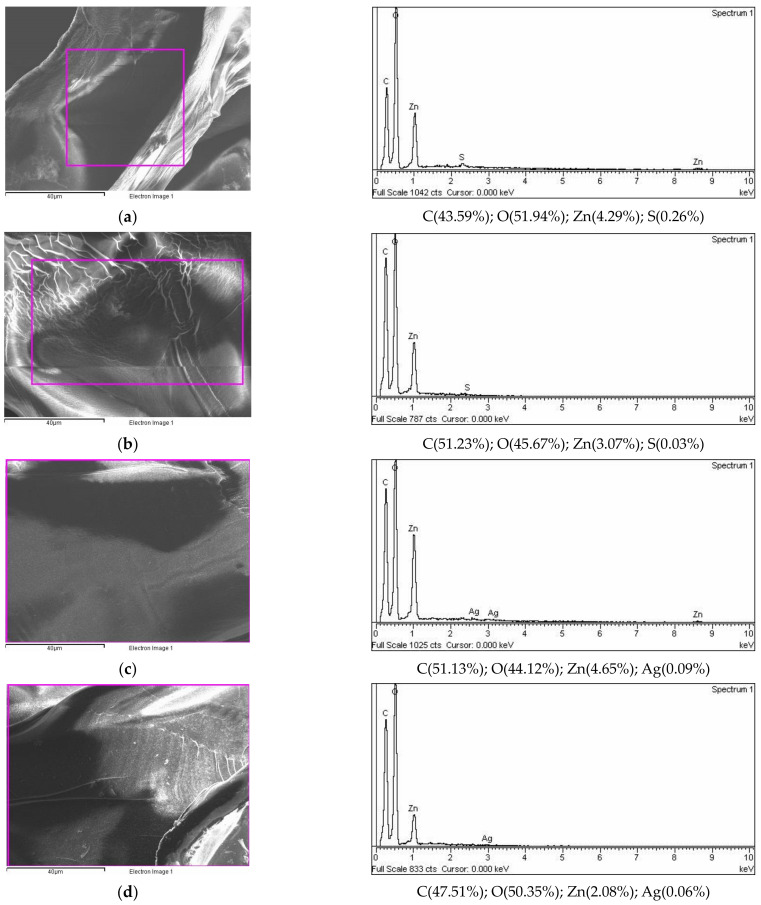
Surface elemental analysis using dispersive X-ray spectroscopy (expressed in the atomic weight percent)) of (**a**) ALG/Zn, (**b**) CS/(ALG/Zn), (**c**) ALG/(Zn+Ag) and (**d**) CS/(ALG/(Zn+Ag)) microparticles. Bars are indicated.

The surface morphology of dried microparticles studied at different SEM magnifications is presented in Figure 2. After drying, all the microparticles were deformed, and the surface became wrinkled with more or less intertwined fibers and pronounced pores. The appearance of wrinkles can be attributed to the loss of water and moisture associated with the stress relaxation processes of biopolymers [43]. The coating of the zinc alginate microspheres with chitosan (Figure 2e) resulted in more pronounced wrinkles with somewhat thicker fibers and waviness. Compared to zinc alginate microspheres with an average pore size of ~69 nm (Figure 3c), the average pore size of microcapsules is smaller (~65 nm) (Figure 2f).

The loading of zinc alginate microspheres with silver showed a surface with thicker fibers and a somewhat higher size of pores (~73 nm) (Figure 2i) compared to zinc alginate. This implies a change in the structure of the gel network due to the silver interactions with alginate and the mechanical influence of cations with a larger ionic radius (the ionic radius of silver ions is 0.126 nm, and that of zinc ions is 0.074 nm). The chitosan layer on the surface of ALG/(Zn+Ag) microspheres is thinner than on the surface of zinc alginate (fibers are thinner and less intertwined) (Figure 2k) with a smaller average pore size (~63 nm) (Figure 2l). The pore size on the surface of the microparticles plays an important role in the release rate of the encapsulated material. The release of encapsulated active substances is faster from microparticles with a larger pore size and vice versa [44].

The EDS spectra analysis of the area nearest to the surface (the electron probe can penetrate to a depth of about 1 μm) exhibited the major percentage of elements corresponding to C and O (Figure 3) The detection of Zn on the surface of the microparticles indicated a part of the Zn is localized near the surface. The small amount of detected S was probably a residue of sulfate used during formulation preparation (cation donor solution—ZnSO_4_). In silver-loaded microparticles, it is also localized near the surface.

**Figure 4:**
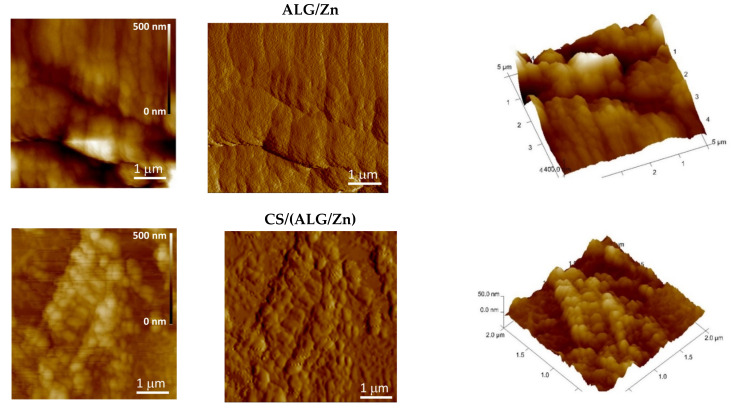

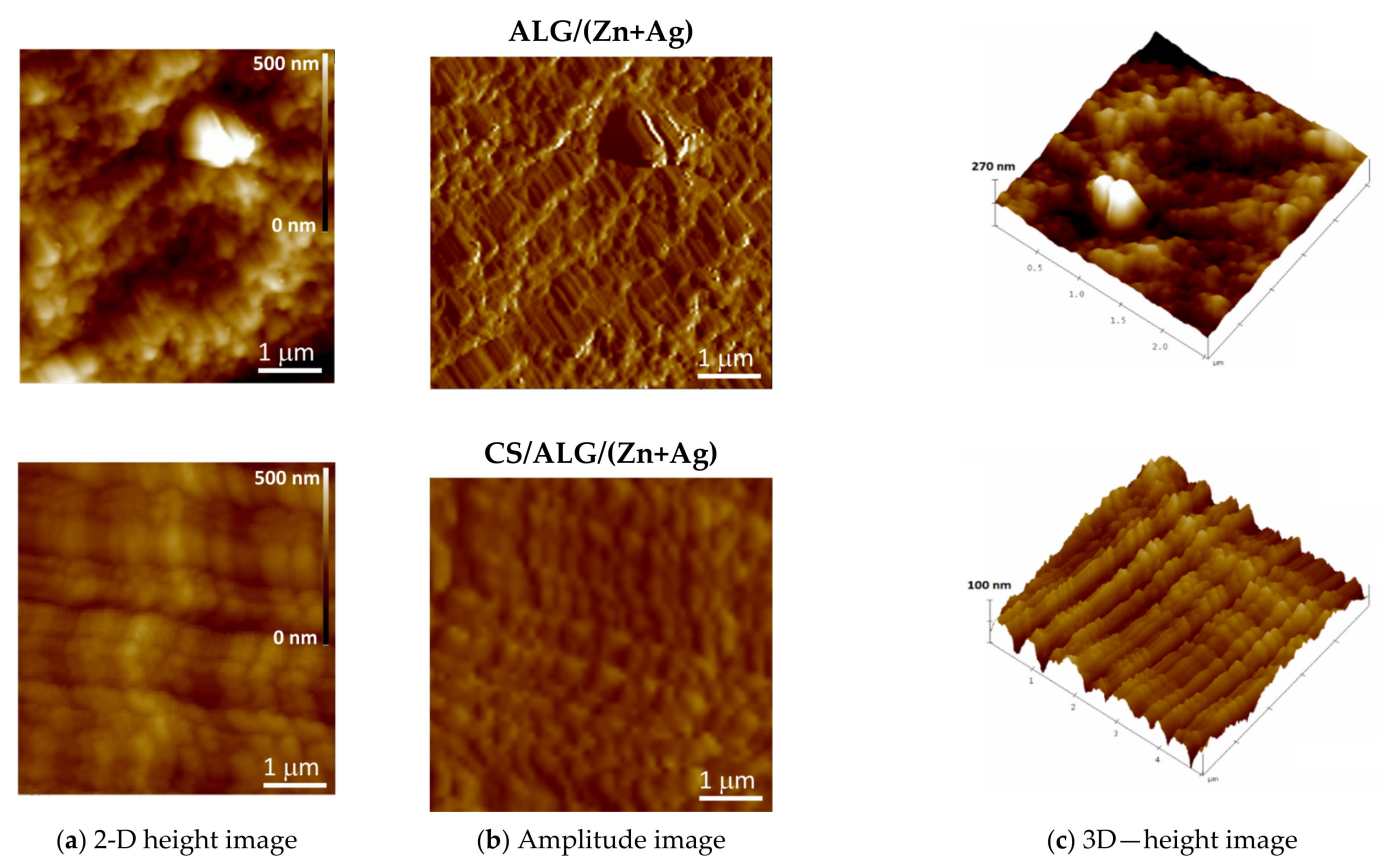
(**a**) 2D—topographic images of height data (top view), (**b**) phase images (top view), and (**c**) 3D topographic images of height data of microparticles as denoted. Bars are indicated.

**Figure 5:**
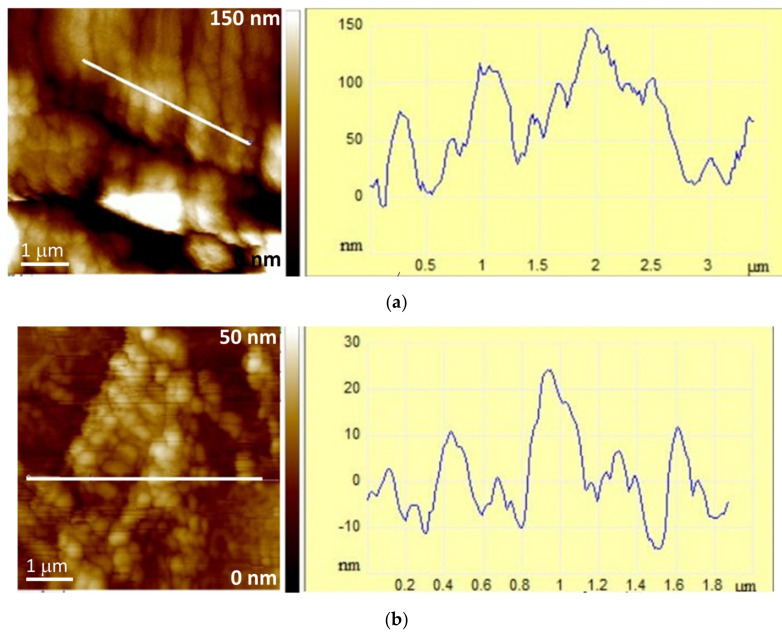

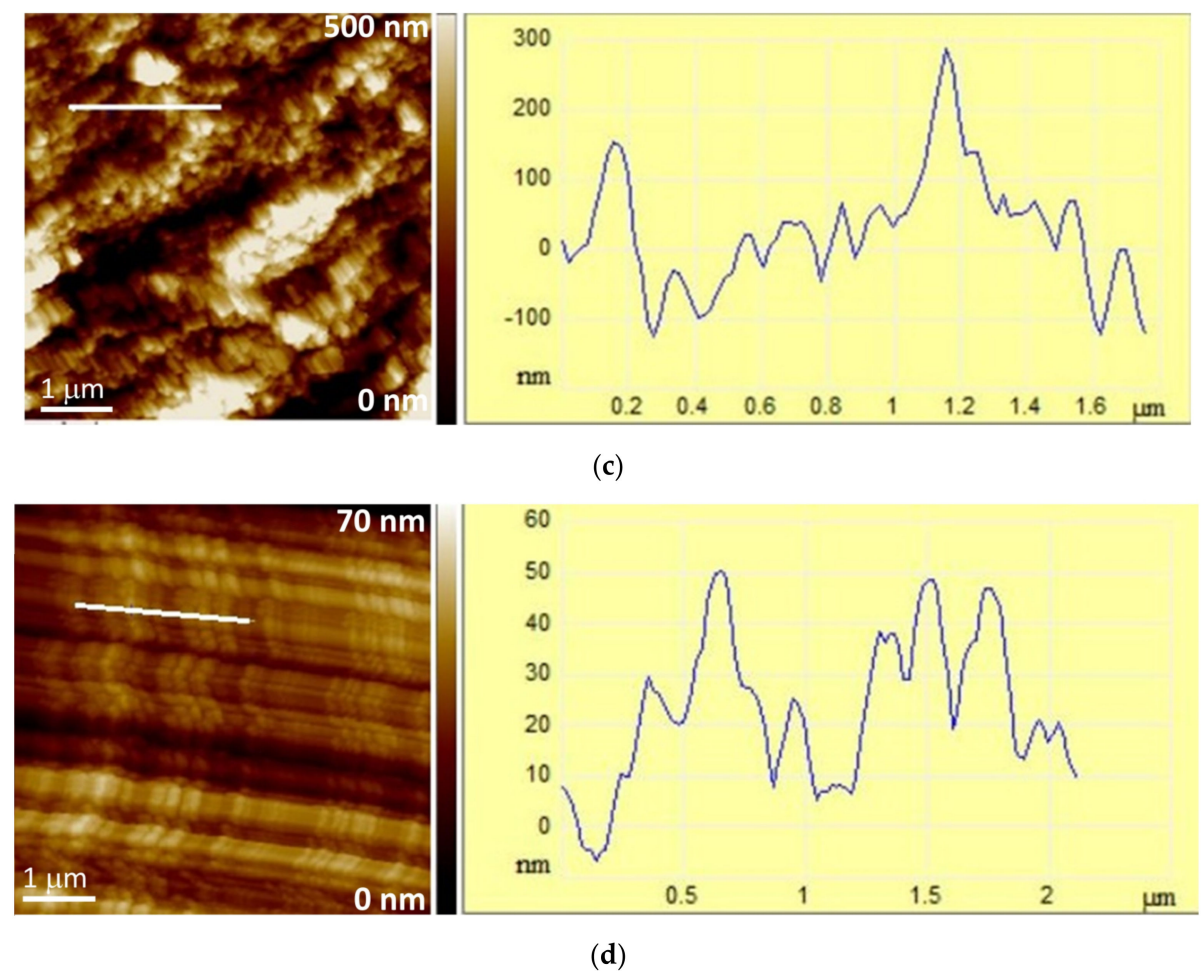
Section analysis profiles (right) along labeled lines (left) of (**a**) ALG/Zn, (**b**) CS/(ALG/Zn), (**c**) ALG/(Zn+Ag), and (**d**) CS/(ALG/(Zn+Ag)) microparticles. Bars are indicated.

The AFM images presented in Figure 4 and Figure 5 complemented the SEM analysis of the surface morphology. The scanned sample area presented by topographic images of height data is shown as the “top view” characterizing individual microparticle morphology and the “3D surface view” with corresponding color scale characterizing the microparticle 3D-height distribution (Figure 4), while the characteristic vertical profile (“section analysis”) of a single microparticle reveals a quantitative 2D height analysis (Figure 5). The ALG/Zn microspheres have granules on the surface that are spatially oriented due to the grain structure and have a regular spherical shape linearly arranged due to the cross-linking of the negatively charged alginate chains by Zn^2+^ ions. A similar structure was already described by Simpliciano et al. [45]. Such crosslinking leads to a high value of roughness (R_a_ = 76 ± 1 nm) (Table 2). The addition of chitosan does not change grain morphology but leads to a loss of directionality and restricts crosslinking to two dimensions, which is expressed in a lower roughness (R_a_ = 7.47 ± 0.02 nm). The addition of Ag+ to ALG/Zn leads to a reduction in the size of the grains, which still have a regular spherical shape, while the degree of lateral crosslinking is reduced and manifests itself in a reduced roughness (R_a_ = 21.97 ± 0.52 nm) in respect to the surface of ALG/Zn (see section analysis profiles in Figure 5). The addition of chitosan to ALG/(Zn+Ag) microspheres only slightly reduces the crosslinking that is still directional, resulting in a slightly lower roughness (R_a_ = 18.62 ± 0.45 nm).

#### 3.1.3. Encapsulation Efficiency, Loading Capacity, Swelling Degree, and Cation Releasing from Microparticles

The determination of encapsulation efficiency (EE) and loading capacity (LC) was performed to obtain information on the relative amount of Zn^2+^ and Ag^+^ ions encapsulated in the microparticles. The results on the loading efficiency and loading capacity of Zn^2+^ and Ag^+^ ions are presented in Table 3. The encapsulation efficiency for Zn^2+^ ions is very high and was almost the same for all types of microparticles. In addition, 100% EE indicated that almost all Ag^+^ ions were encapsulated. The addition of chitosan decreased the loading capacity of microcapsules in comparison to microspheres due to the diffusion in the media during the microcapsule process preparation.

When dispersed in solution, microparticles swell, thus changing several properties, including mechanical strength, permeability, release behavior, stability, and the rate of disintegration. The swelling process involves two underlying molecular processes: (i) penetration of the solution into the matrix and (ii) relaxation of polymer stress (transition of glassy structure to a rubbery state) [46]. The greater extent of swelling of microparticles simultaneously loaded with Zn^2+^ and Ag^+^ ions than those loaded only with Zn^2+^ ions may be attributed to the less dense alginate network structure due to the addition of Ag^+^ ions. During gelation of sodium alginate, Zn^2+^ cations interact with carboxylate groups forming a crosslinked network of alginate chains. Ag^+^ ion addition and concomitant gelation likely differ from those with Zn^2+^ ions, thus causing changes in the properties of the gel network. The extent of crosslinking determines the density of the hydrogel that affects its swelling. It was shown that swelling can be used to determine the degree of crosslinking [47]. When a microsphere is dispersed in a medium, a higher degree of crosslinking and a denser network structure result in less swelling and, as a result, lower S_w_ values. Compared to microspheres, those coated with chitosan (microcapsules) exhibit a higher degree of swelling. This can be attributed to the higher water uptake capabilities of chitosan [48,49]. The encapsulation efficiency, loading capacity, and swelling dynamics of microparticles emphasize the importance of Zn^2+^ and Ag^+^ ions and the incorporation of chitosan in modulating these properties.

**Figure 6:**
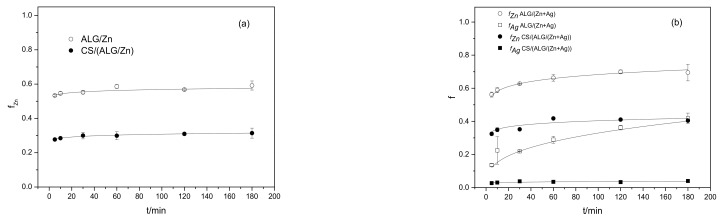
(**a**) Fraction of the Zn^2+^ (f_Zn_) released from ALG/Zn and CS/(ALG/Zn) microparticles and (**b**) fraction of released Zn^2+^ (f_Zn_) and Ag^+^ (f_Ag_) released from ALG/(Zn+Ag) and CS/(ALG/(Zn+Ag)) microparticles with time (t).

The possible use of biopolymer microparticles Imposes research on their release capacity in certain physicochemical conditions. The release profiles of Zn^2+^ and Ag^+^ ions from different types of alginate microparticles are presented in Figure 6a,b. A set of release profiles exhibited a burst initial release followed by a slower release obeying the power law equation. It can be seen that the amount of cations released depends on the active agents and the presence of chitosan. The release patterns of Zn^2+^ and Ag^+^ ions heavily rely on the presence of chitosan, which had a profound influence on the release rates.

All curves presented in Figure 6a,b can be described by the equation:

f = *k*t*^n^* (5)

where f represents the fraction of released cations, *k* is a constant characteristic of the active agent/polymer system that considers structural and geometrical aspects of the system, and the exponent *n* characterizes the mechanism controlling the release of active agents from microparticles. The values of the release constants *k* and the exponent *n* are listed in Table 4. The correlation coefficients ranged from 0.97 to 0.99.

Various mechanisms such as desorption from the surface, diffusion through the microparticle matrix and wall, and microparticle disintegration, dissolution, or erosion of the structure, or their combination, may be included in the release of active agents from microparticles. The most important release mechanisms of hydrophilic microparticles are swelling and dissolution/erosion at the microcapsule surface [46]. When dispersed in deionized water, the hydrophilic polymer microcapsules swelled, thus influencing the release of cations from them. To identify the type of rate-controlling mechanism involved in cations release, a semi-empirical Korsmeyer–Peppas model was applied [50]. According to Korsmeyer–Peppas, the release exponent *n* can be characterized by three different mechanisms (Fickian diffusion, anomalous (non-Fickian diffusion), or Type II transport). Values of *n* < 0.43 indicate that the release is controlled by classical Fickian diffusion, *n* > 0.85 is controlled by Type II transport, involving swelling of the polymer and relaxation of the polymeric matrix, while values of *n* between 0.43 and 0.85 show the anomalous transport kinetics determined by a combination of the two diffusion mechanisms and Type II transport. Lower *n* values than 0.43 for all microparticles (Table 4) indicated that the rate-controlling release mechanism involved is a classical Fickian diffusion. The results showed that changing the extent of crosslinking did not affect the release control mechanism.

The *k* values pointed out remarkable differences in the release rate of Zn^2+^ ions between microspheres and microcapsules. This can be ascribed to the coating of the microspheres with a polyelectrolyte layer and differences in the structure of the microparticles. In addition to the mechanical barrier and the smaller pores, chitosan can bind metal ions to amino (-NH_2_) and to a lesser extent via hydroxyl (–OH) groups [51]. A significantly lower proportion of released silver ions can be explained by slower diffusion through the microcapsule matrix and probably greater binding to chitosan than to zinc ions. A comparison of our results with data from the literature showed that the concentration of Zn^2+^ and Ag^+^ ions released from prepared microparticle formulations in deionized water is below the level of plant toxicity [10].

### 3.2. Antifungal Effect of Microparticles on the B. cinerea Growth

The antifungal effect of microparticles on *B. cinerea* growth was followed after 5 days. Because CS/(ALG/Zn) microcapsules did not exhibit an inhibitory effect (the growth and sporulation were the same as in the control), the results are not shown. Despite the antifungal properties of chitosan [12,14,52], it appears that the amount of chitosan and released Zn^2+^ ions was not enough to cause an inhibitory effect during observation (5 days). However, three other microparticle types showed substantial inhibitory effects with variations in the degree of inhibition based on the amount of microparticles used.

**Figure 7:**
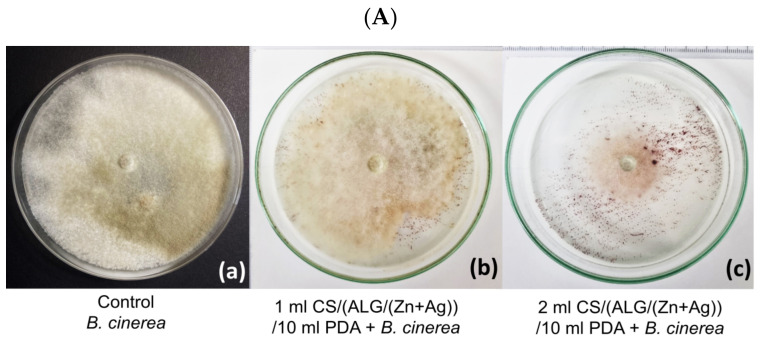

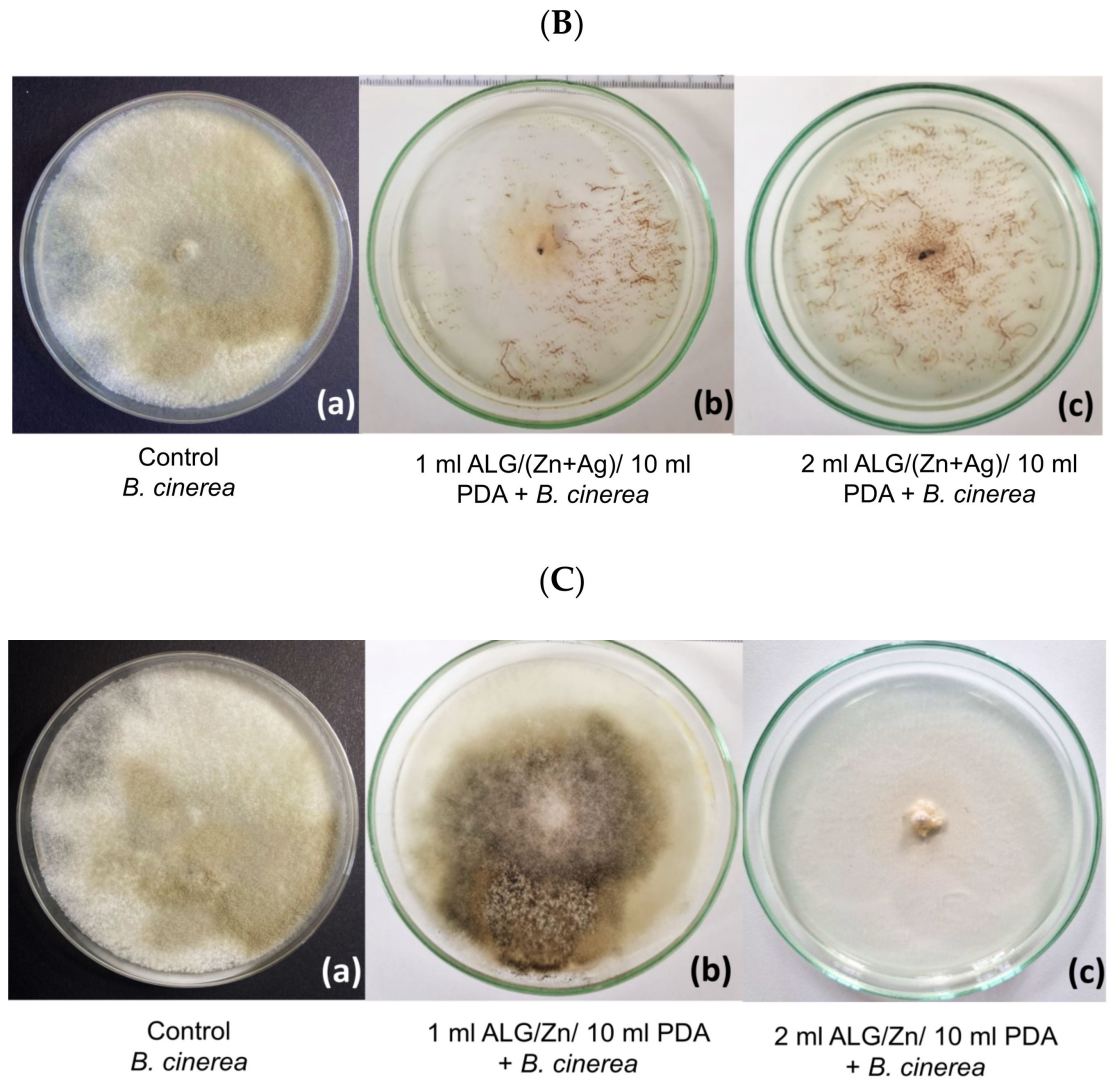
Antifungal effect of (**A**) CS/(ALG/(Zn+Ag)), (**B**) ALG/(Zn+Ag), and (**C**) ALG/Zn microparticles on the growth of *Botrytis cinerea* after 5 days. *B. cinerea* was inoculated into PDA medium using a micellar disk (Ø 5 mm); the diameter of the Petri dishes was 9 cm; (**a**) *B. cinerea* on PDA; (**b**–**c**) *B. cinerea* on PDA with microparticles.

**Figure 8:**
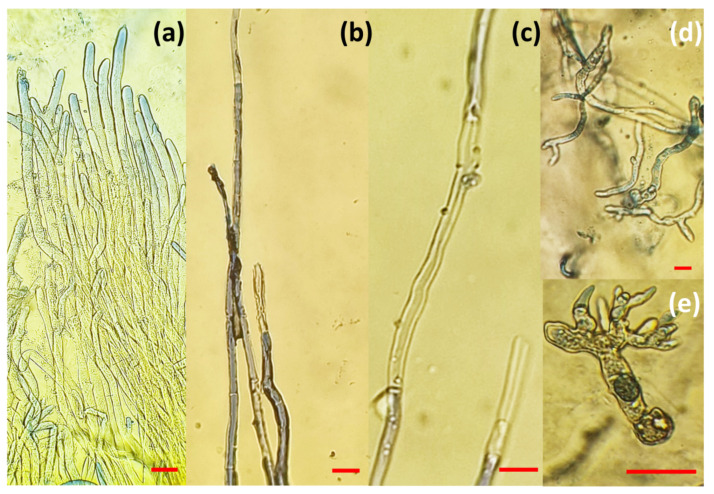
Microscopic changes in hyphae of the pathogen *B. cinerea* after five days of growth in medium with (**b**) CS/(ALG/(Zn+Ag)) microcapsules, (**c**) ALG/(Zn+Ag) microspheres, (**d**,**e**) ALG/Zn microspheres and (**a**) denotes control sample. All bars denote 10 µm.

The inhibitory effects of the other three types of microparticles are presented in Figure 7 and Figure 8 and listed in Table 5 and Table 6. All microparticles tested were significantly effective in inhibiting *B. cinerea* although the effects differed in the degree of inhibition of the pathogen. In variants with a smaller volume of microparticles in medium (1 mL/10 mL PDA), the average growth of *B. cinerea* was less inhibited than in variants with a larger volume of microparticles. Inhibition in all variants is significant compared to control (Figure 7(Ac)) (Table 5 and Table 6). The inhibition effect increases in order CS/(ALG/(Zn+Ag)) < ALG/Zn < ALG/(Zn+Ag).

Figure 8 presents microscopic changes in hyphae of the pathogen *B. cinerea* after five days of growth on a medium with microparticles. Figure 8a shows the mycelium from the edge of *B. cinerea* culture in control (untreated samples), showing hyphae with a typical “reticulated” structure and a smooth surface, while the hyphae were turgid and regular. After treatment with microparticles in two variants, the hyphae lost their smoothness and formed unusual protrusions on the surface of the fungal hyphae, indicating that the microparticles inhibited the growth of *B. cinerea* by deforming the structure of the hyphae. After treatment, sporulation was also reduced.

Hyphae deformations were more significant in the variants that contained 2 mL of medium. An unusual pattern of hyphal growth and deformation was particularly observed in treatment with ALG/Zn microspheres (Figure 8d,e), while in the treatment containing a combination of zinc and silver ions, that is, with ALG/(Zn+Ag) microspheres, the hyphae were empty and had a degraded cell wall (Figure 8c). Hyphae treated with CS/(ALG/(Zn+Ag)) microcapsules (Figure 8b) show a “melted” appearance and loss of cell wall stability, although the contents within the hyphae were present. Compared to the control, sporulation was partially reduced.

These results suggest that treatments with three types of microparticles can significantly inhibit the growth of *B. cinerea*. The most effective were ALG/(Zn+Ag) microspheres applied in a volume of 2 mL, which suppressed *B. cinerea* in a significant percentage of 98.9% and also led to a reduction in sporulation. Another significant treatment was the treatment with ALG/Zn microspheres, which suppressed the pathogen by 92.3%. The CS/(ALG/(Zn+Ag)) microcapsules have a weaker antifungal effect than the other variants, presumably because these microparticles release the ions in the substrate more slowly. Because *B. cinerea* is a fast-growing pathogen, the ions from these microparticles could not be released quickly enough to suppress *B. cinerea* by a significant percentage. This is confirmed by the remaining variants without chitosan, where the release of ions in the substrate was significantly faster, which is confirmed by the high levels of inhibition. Since the method was used where the fungus directly absorbs components from the substrate, in the version with chitosan, the action of the inhibitory ions was delayed, which allowed the pathogen to develop. The results are consistent with the results of various studies that confirm the significant suppression of pathogenic microorganisms treated with metal ions [53,54,55]. The degrees of suppression depend on the composition of the microparticles, their combination with other elements, and the tolerance of the target pathogen, as well as the amount of released cations (Figure 6a,b).

Some studies have shown that particles with zinc ions can cause structural changes in the membrane of microbial cells, causing cytoplasmic leakage and ultimately the death of bacterial cells [56,57], i.e., that the integration of ions into cells can induce the continuous release of membrane lipids and proteins, which changes the membrane permeability of pathogen [58,59]. The results obtained from the microscopic analysis showed activity on the cell membrane of the fungus, which is a result following the review of the literature. Interestingly, the mechanism of the inhibitory action of zinc ions on microorganisms is not fully understood. Our results suggest that ALG/Zn microspheres can affect cell functions and ultimately cause an increase in nucleic acid content, due to the stress response of fungal hyphae in the form of self-protection against microspheres [55,59]. Swollen hyphae were also observed in the treatment with ALG/(Zn+Ag) microspheres in this study (Figure 8e), and this has been reported by others [55,60,61]. Such data suggest a different mechanism of the inhibitory effect of zinc ions on fungi compared to those reported earlier for bacteria [55,62].

Likewise, the successful inhibition of *B. cinerea* using silver ions was proven by Jo et al. [63]. An increased inhibitory effect of silver nanoparticles against fungal spores compared to hyphal growth was reported, which can be attributed to structural differences between spores and vegetative walls of fungi. The chitin content of many fungal species is significantly higher in hyphal walls compared to spore walls, making them more sensitive to heavy metals [64] which is evidence of why hyphae are the target site. Furthermore, during the spore germination, enzymes such as disulfide reductases and glucanases result in the softening of the cell walls, to facilitate germination, and elongation of the tubes and thus create sensitive sites for toxic substances to contact the fungal cell [65]. This increased toxic effect against fungal spores, compared to hyphal growth, can be attributed to structural differences between the spores and the walls of vegetative fungi. The chitin content of many fungal species is significantly higher in the hyphal walls compared to the spore walls, which makes them more sensitive to heavy metals. Furthermore, during the process of spore germination, enzymes such as disulfide reductases and glucanases result in the softening of cell walls, to facilitate germination, and elongation of the tubes and thus create sensitive sites for toxic substances when they come into contact with the fungal cell [55,64]. Silver is known to attack a wide range of biological processes in microorganisms, including cell membrane structure and function [66]. Silver also inhibits the expression of proteins associated with ATP production [67]. Previous reports indicated that the antimicrobial activity of silver varied depending on the type of microbe [68]. Silver nanoparticles can significantly delay mycelial growth in a dose-dependent manner in vitro [69] and can directly bind to and penetrate the cell membrane to reduce spores, although the penetration of silver nanoparticles into microbial cell membranes is not fully understood [70].

Ionic or nanoparticle silver’s antifungal action offers a lot of potential for application in the management of spore-producing fungal plant diseases. Silver might not be as harmful to people and animals as synthetic fungicides [63]. Several commercially accessible agricultural bio-/pesticides have been developed using zinc alone or in conjunction with copper. The antibacterial efficacy of zinc formulations against a variety of phytopathogens has gained popularity. Zinc formulations with antibacterial properties can be utilized as a low-cost, ecologically friendly, and effective microbicide with a range of activities such as bactericide, fungicide, or algaecide, among others [71]. This study also suggests that ALG/(Zn+Ag) microparticles were more effective than ALG/Zn microparticles in controlling a fast-growing pathogen. Due to the slower release of active substances, CS/(ALG/Zn+Ag)) microcapsules probably could be used to protect plants during the entire growing season. Therefore, microparticles prepared by a cost-effective method hold great promise as an antimicrobial agent and have antifungal activity with great potential for use in the control of spore-producing fungal plant pathogens. Several parameters will require evaluation before practical application, including phytotoxicity and antimicrobial effects and the development of microparticle delivery systems to host tissues colonized by phytopathogens [72].

## 4. Conclusions

The structural complexity of ALG/Zn microparticles, influenced by different factors such as the addition of silver ions, chitosan, and conditions like the drying process, has been characterized in detail. The alterations in microparticle size, surface morphology, pore size, and roughness emphasize their potential influence on the release rate of encapsulated metal ions. Furthermore, the surface elemental composition highlighted the localization of certain elements providing insights into the interaction dynamics within the formulation. Hydrogen bonding and electrostatic interactions were found to be prevalent among the microparticle constituents. Metal ions and the incorporation of chitosan modulate encapsulation efficiency, loading capacity, and swelling dynamics of microparticles. The encapsulation efficiencies for both metal ions were found to be commendably high. Distinct differences in the swelling behaviors between microparticles were primarily attributed to the gel network structure and the presence of chitosan, highlighting its role in enhancing water uptake capabilities.

Moreover, the release patterns of metal ions demonstrated that the presence of chitosan had a profound influence on the release rates, dictated primarily by a Fickian diffusion mechanism. Significant differences in the release behavior between zinc and silver ions depend on the interactions between the constituents of the microparticles (electrostatic interactions, hydrogen bonding and complexation), the degree of cross-linking, pore size and surface roughness, the difference in the size of the ionic radius between the ions, as well as the presence of a chitosan layer on the surface of microparticles. The findings offer a comprehensive understanding of the microparticle system, laying the groundwork for potential optimization and tailoring for specific applications.

The inhibitory effects on the growth of *B. cinerea* of three types of microparticles were substantial, and the degree of inhibition varied depending on the amount of microparticles used and metal ions released. Zinc alginate microspheres and silver-loaded zinc alginate microparticles were significantly effective in inhibiting the growth of *B. cinerea* although the effects differed in the degree of pathogen inhibition. The inhibition effect increases in the order CS/(ALG/(Zn+Ag)) < ALG/Zn < ALG/(Zn+Ag). Since *B. cinerea* is a fast-growing pathogen, the ions from zinc alginate microcapsules cannot be released fast enough to control *B. cinerea* in a significant percentage, indicating that this formulation appears to be suitable for plant protection during the growing season.

These results point towards the potential of these microparticles as promising antifungal agents, especially for spore-producing fungal plant pathogens. Further evaluations, including phytotoxicity and antimicrobial effects, are needed for practical application.

## Figures and Tables

**Table 1:** Average size (d/µm) of wet and lyophilized ALG/Zn and ALG/(Zn+Ag) microspheres and CS/(ALG/Zn) and CS/(ALG/(Zn+Ag)) microcapsules obtained by light microscopy.

Microparticles	d_wet_	d_dry_
ALG/Zn	681.06 ± 92.52 ^c^	381.54 ± 41.27 ^c^
CS/(ALG/Zn)	852.17 ± 47.35 ^b^	515.50 ± 1.93 ^b^
ALG/(Zn+Ag)	915.46 ± 23.88 ^b^	598.24 ± 6.85 ^a^
CS/(ALG/(Zn+Ag))	1112.48 ± 33.81 ^a^	557.52 ± 2.03 ^ab^

^a–c^ = differences between the values within a column (values not superscripted with the same letter are statistically different, Tukey post-test, *p* < 0.05).

**Table 2:** Roughness parameters, average roughness (R_a_), root mean square of roughness (R_q_), and Z range for analyzed samples.

Microparticles	R_a_/nm	R_q_/nm	Z Range/nm
ALG/Zn	76 ± 1 ^a^	106 ± 2 ^a^	1127 ± 21 ^a^
CS/(ALG/Zn)	7.47 ± 0.02 ^d^	9.20 ± 0.08 ^d^	53.09 ± 0.67 ^d^
ALG/(Zn+Ag)	21.97 ± 0.52 ^b^	28.88 ± 0.74 ^b^	248.33 ± 0.96 ^b^
CS/(ALG/(Zn+Ag)	18.62 ± 0.45 ^c^	23.19 ± 0.68 ^c^	181 ± 2 ^c^

^a–d^ = differences between the values within a column (values not superscripted with the same letter are statistically different, Tukey post-test, *p* < 0.05).

**Table 3:** Encapsulation efficiency (EE/%), loading capacity (LC/mg g^−1^), and degree of swelling (S_w_/%) of microparticles.

Microparticles	EE_Zn_	EE_Ag_	LC_Zn_	LC_Ag_	S_w_
ALG/Zn	87.21 ± 0.49 ^a^	-	14.19 ± 0.83 ^a^	-	15.43 ± 3.82 ^d^
CS/(ALG/Zn)	87.21 ± 0.49 ^a^	-	8.91 ± 0.11 ^b^	-	43.8 ± 1.65 ^b^
ALG/(Zn+Ag)	87.32 ± 0.40 ^a^	99.99 ± 0.001 ^a^	15.27 ± 0.83 ^a^	19.48 ± 0.50 ^a^	25.12 ± 0.11 ^c^
CS/(ALG/(Zn+Ag))	87.32 ± 0.40 ^a^	99.99 ± 0.001 ^a^	7.71 ± 0.52 ^b^	4.22 ± 0.47 ^b^	66.33 ± 0.45 ^a^

^a–d^ = differences between the values within a column (values not superscripted with the same letter are statistically different, Tukey post-test, *p* < 0.05).

**Table 4:** Variation of the release constant (*k*/min), exponent (*n*) of Zn^2+^ and Ag^+^ ions released from microparticles.

Microparticles	*k*_Zn_	*n*_Zn_	*k*_Ag_	*n*_Ag_
ALG/Zn	0.51	0.02	-	-
CS/(ALG/Zn)	0.26	0.03	-	-
ALG/(Zn+Ag)	0.50	0.07	0.07	0.06
CS/(ALG/(Zn+Ag))	0.31	0.06	0.02	0.07

**Table 5:** Antifungal effect of different types of microparticles (1 mL/10 mL PDA) on the growth of *Botrytis cinerea* after 5 days.

	Control		Antifungal Test	
	1 mL dH_2_O/10 mL PDA + *B. cinerea*	1 mL CS/(ALG/(Zn+Ag)) /10 mL PDA + *B. cinerea*	1 mL ALG/(Zn+Ag) /10 mL PDA + *B. cinerea*	1 mL ALG/Zn /10 mL PDA + *B. cinerea*
$\bar{x}$ (cm^2^) ± SD	57.5 ± 0.2 ^a^	41.7 ± 1.9 ^b^	10.1 ± 1.3 ^c^	37.2 ± 2.0 ^d^
I (%)	0%	27.5%	82.5%	35.4%

different letters indicate a statistically significant difference between mean values within the concentration range (Tukey test, *p* < 0.05).

**Table 6:** Antifungal effect of different types of microparticles (2 mL/10 mL PDA) on the growth of *Botrytis cinerea* after 5 days.

	Control		Antifungal Test	
	2 mL dH_2_O/10 mL PDA *B. cinerea*	2 mL CS/(ALG/(Zn+Ag)) /10 mL PDA + *B. cinerea*	2 mL ALG/(Zn+Ag) /10 mL PDA + *B. cinerea*	2 mL ALG/Zn /10 mL PDA + *B. cinerea*
$\bar{x}$ (cm^2^) ± SD	56.2 ± 0.4 ^a^	13.6 ± 0.8 ^b^	0.6 ± 1.3 ^c^	4.3 ± 1.9 ^c^
I (%)	0%	75.8%	98.9%	92.3%

^a,b,c^ different letters indicate a statistically significant difference between mean values within the concentration range (Tukey test, *p* < 0.05).

## Data Availability

Data are contained within the article.
